# Constitutive hyperproduction of sorbicillinoids in *Trichoderma reesei* ZC121

**DOI:** 10.1186/s13068-018-1296-4

**Published:** 2018-10-25

**Authors:** Chengcheng Li, Fengming Lin, Wei Sun, Shaoxun Yuan, Zhihua Zhou, Fu-Gen Wu, Zhan Chen

**Affiliations:** 1 State Key Laboratory of Bioelectronics, School of Biological Science and Medical Engineering, Southeast University, Nanjing, 210096 China; 2 Key Laboratory of Synthetic Biology, Institute of Plant Physiology and Ecology, Shanghai Institutes for Biological Sciences, Chinese Academy of Sciences, Shanghai, 200032 China; 3 Department of Chemistry, University of Michigan, 930 North University Avenue, Ann Arbor, MI 48109 USA; 4 Nanjing, China

**Keywords:** Biosynthetic gene cluster, Secondary metabolites, Natural product, Yellow pigment, Sorbicillinoids, *Trichoderma reesei*

## Abstract

**Background:**

In addition to its outstanding cellulase production ability, *Trichoderma reesei* produces a wide variety of valuable secondary metabolites, the production of which has not received much attention to date. Among them, sorbicillinoids, a large group of hexaketide secondary metabolites derived from polyketides, are drawing a growing interest from researchers because they exhibit a variety of important biological functions, including anticancer, antioxidant, antiviral, and antimicrobial properties. The development of fungi strains with constitutive, hyperproduction of sorbicillinoids is thus desired for future industry application but is not well-studied. Moreover, although *T. reesei* has been demonstrated to produce sorbicillinoids with the corresponding gene cluster and biosynthesis pathway proposed, the underlying molecular mechanism governing sorbicillinoid biosynthesis remains unknown.

**Results:**

Recombinant *T. reesei* ZC121 was constructed from strain RUT-C30 by the insertion of the gene 12121-knockout cassette at the telomere of *T. reesei* chromosome IV in consideration of the off-target mutagenesis encountered during the unsuccessful deletion of gene 121121. Strain ZC121, when grown on cellulose, showed a sharp reduction of cellulase production, but yet a remarkable enhancement of sorbicillinoids production as compared to strain RUT-C30. The hyperproduction of sorbicillinoids is a constitutive process, independent of culture conditions such as carbon source, light, pH, and temperature. To the best of our knowledge, strain ZC121 displays record sorbicillinoid production levels when grown on both glucose and cellulose. Sorbicillinol and bisvertinolone are the two major sorbicillinoid compounds produced. ZC121 displayed a different morphology and markedly reduced sporulation compared to RUT-C30 but had a similar growth rate and biomass. Transcriptome analysis showed that most genes involved in cellulase production were downregulated significantly in ZC121 grown on cellulose, whereas remarkably all genes in the sorbicillinoid gene cluster were upregulated on both cellulose and glucose.

**Conclusion:**

A constitutive sorbicillinoid-hyperproduction strain *T. reesei* ZC121 was obtained by off-target mutagenesis, displaying an overwhelming shift from cellulase production to sorbicillinoid production on cellulose, leading to a record for sorbicillinoid production. For the first time, *T. reesei* degraded cellulose to produce platform chemical compounds other than protein in high yield. We propose that the off-target mutagenesis occurring at the telomere region might cause chromosome remodeling and subsequently alter the cell structure and the global gene expression pattern of strain ZC121, as shown by phenotype profiling and comparative transcriptome analysis of ZC121. Overall, *T. reesei* ZC121 holds great promise for the industrial production of sorbicillinoids and serves as a good model to explore the regulation mechanism of sorbicillinoids’ biosynthesis.

**Electronic supplementary material:**

The online version of this article (10.1186/s13068-018-1296-4) contains supplementary material, which is available to authorized users.

## Background

Secondary metabolites are structurally heterogeneous, highly bioactive, and low-molecular weight compounds synthesized by bacteria, fungi, algae, plants, and animals [1–5]. Unlike primary metabolites, they are not directly essential for the growth of the corresponding production organism but can broaden the inhabitable environments or beat other competitive organisms in a given ecological niche [6]. Most secondary metabolites are derived from either nonribosomal peptides (NRPs) or polyketides (PKSs) or both mixed together, whereas others are derived from terpenes or fatty acids [7]. These molecules include important pharmaceuticals (e.g., penicillin, cyclosporin and statins), potent toxins (e.g., aflatoxins and trichothecenes) and Janus-faced compounds (e.g., ergot alkaloids), holding both economical and health implications for humans [8].

Sorbicillinoids, also termed “yellow pigment,” are derived from PKS and a large group of hexaketide secondary metabolites and include the cyclization on the carboxylate terminus. Sorbicillinoids are produced and secreted by both marine and terrestrial ascomycetes, including *Trichoderma* [9], *Aspergillus* [10], *Penicillium* [11], *Streptomyces* [1], *Acremonium* [12], *Paecilomyces* [13], and *Eurotiomycete* [14]. Most of these compounds possess the characteristic C1–C6 sorbyl sidechain and bi- or tri-cyclic frameworks that are extremely complex and highly oxygenated. Based on their structure, sorbicillinoids fall into four classes: monomeric sorbicillinoids, bisorbicillinoids, trisorbicillinoids and hybrid sorbicillinoids. They have a variety of biological activities, including anticancer [15], antioxidant [16], antiviral [17] and antimicrobial [18], showing promising applications in the agriculture, pharmaceutical, and food industries. Therefore, these yellow pigments have attracted considerable interest. Historically, however, most work was aimed at eliminating yellow pigments from fungal fermentation cultures for the production of products like β-lactams [19] and cellulase [9].

Studies of sorbicillinoids have been performed primarily with *Trichaderma* [9] and *Penicillium* species [11]. As a well-known industrial strain for the production of cellulases, hemicellulases, and recombinant proteins [20], *T. reesei* is also a rich source of secondary metabolites [21, 22], a point which often is overlooked. It has been observed that *T. reesei* forms yellow pigments during growth [23, 24], which have been identified as a mixture of sorbicillin, sorbicillinol and sorbicillinoids [9, 25, 26]. More recently, the biosynthesis pathway of sorbicillinoids in *P. chrysogenum* [27] and *T. reesei* [25] has been proposed, wherein all the related genes are clustered on the genome the same way as most genes involved generally in secondary metabolites production [9, 19, 25]. This cluster also contains two transcriptional factors, YPR1 and YRP2, and a transporter [9]. Still, the biosynthesis mechanism of sorbicillinoids remains obscure and strain improvement for sorbicillinoids’ hyperproduction has not been reported.

In this study, the recombinant *T. reesei* strain ZC121 was obtained by off-target mutagenesis resulting from the unsuccessful deletion of gene 121121. Strain ZC121 was found to produce only a small amount of cellulase and hemicellulase, but a record yield of sorbicillinoids. The effect of culture conditions on this sorbicillinoids’ hyperproduction was determined, including carbon source, light, pH and temperature. Furthermore, identification of the off-target mutagenesis, phenotype profiling and comparative transcriptional profiling were carried out to reveal the molecular mechanism(s) underlying sorbicillinoid hyperproduction of ZC121.

## Materials and methods

### Materials

Construction and propagation of plasmids were performed in *Escherichia coli* DH5α. *Agrobacterium tumefaciens* AGL-1 served as a T-DNA donor for transformation of *T. reesei* RUT-C30 (CICC 13052) [28]. *E. coli* DH5α and *A. tumefaciens* AGL-1 were cultured in Luria–Bertani (LB) with 220 rpm at 37 °C and 28 °C, respectively. *T. reesei* RUT-C30 and its derivatives were cultured on potato dextrose agar (PDA) plates at 28 °C with mixing at 200 rpm for conidia production and in *Trichoderma* minimal media (TMM) [29] with 2% (w/t) cellulose or other carbon sources (as indicated) for cellulase and sorbicillinoid production. Plasmid pXBthg was employed to construct plasmid pXBthg-121121 (Additional file 1: Fig. S1) [30]. The primers used in this study can be found in Additional file 1: Table S1. Fifty μg/mL of hygromycin B was utilized as the selection marker. All chemicals used in this study were purchased from Sigma-Aldrich, USA.

### Strain construction

Genomic DNA was extracted from *T. reesei* cells grown in sabouraud dextrose broth (SDB) medium for 48 h at 28 °C using the E-Z 96 Fungal DNA Kit (Omega Bio-tek, Germany). The 1500-bp upstream or downstream region abutting gene 121121 (Additional file 1: Fig. S2) were amplified from the prepared DNA template, respectively. The 1500-bp downstream fragment was cloned into plasmid pXBthg at *Bam*HI using ClonExpress™ II One Step Cloning Kit (Vazyme, China), which was followed by the cloning of the 1500-bp upstream one at XhoI, resulting in the plasmid pXBthg-121121 (Additional file 1: Fig. S1). pXBthg-121121 was then introduced into *T. reesei* RUT-C30 by the *Agrobacterium tumefaciens*-mediated transformation (AMT) method [28]. Four transformants ZC121-1, ZC121-2, ZC121-3, and ZC121-4 were obtained after selection on PDA plates containing 50 μg/mL hygromycin B and 200 μM cefotaxime.

### Shake flask cultivation

Five percent (v/v) 10^7^/mL conidia from *T. reesei* grown on PDA plates at 28 °C for 7 days were inoculated into 10 mL SDB and incubated at 28 °C with mixing at 200 rpm for 2 days. Ten percent (v/v) pre-grown mycelia were inoculated into 50 mL TMM media (pH 6) with 2% cellulose, lactose, glucose, galactose, or glycerol, and then incubated at 28 °C with mixing at 200 rpm for 5 days. A 0.5 mL culture sample was taken every 12 h. The samples were centrifuged at 14,000×*g* for 10 min at 4 °C and the supernatants were filtrated with 0.22 µm filter membranes and stored at − 80 °C for sorbicillinoid analysis and the cellulase activity assay.

The absorbance at 370 nm of the prepared supernatant as mentioned above was recorded using a UV spectrophotometer to determine the amount of sorbicillinoids present [31]. To determine the exact yield of sorbicillinoids, the prepared supernatant was dried at 40 °C overnight. Methanol was used to extract the sorbicillinoids from the dried supernatant powder several times until the powder became white. The supernatant containing sorbicillinoids in methanol was collected after centrifuging and methanol was removed by rotary evaporation. The crude sorbicillinoids product was then obtained and utilized to determine the standard curve (Additional file 1: Fig. S3) for the yield calculation of sorbicillinoids.

The composition of the crude sorbicillinoids product from *T. reesei* ZC121 and RUT-C30 were analyzed using LC–MS system (G2-XS QTof, Waters) coupled with a UPLC column (2.1 × 100 mm ACQUITY UPLC BEH C18 column containing 1.7 μm particles). Reserpine was used as the internal standard [32]. Two microlitre samples dissolved in methanol were injected onto the C18 column at a flow rate of 0.4 mL/min. The gradient for elution was 2% buffer B (0.1% formic acid in water) for 0.5 min, 2–20% buffer B for 5 min, 20–95% buffer B for 6 min, and finally 95% buffer B for 2 min. Mass spectrometry was operated using the electrospray source in positive ion mode with MSe acquisition according to the following settings: the selected mass ranged from 50 to 1200 m/z and leucine enkephalin (m/z 556.2771) was used as the “lock mass option” for recalibration. The ionization parameters were set as follows: capillary voltage was 2.5 kV, collision energy was 40 eV, source temperature was 120 °C, and the desolvation gas temperature was 400 °C. Data acquisition and processing were performed using Masslynx 4.1.

### Analysis methods

For cellulase activity assay, biomass measurement, RT-qPCR analysis and whole genome resequencing, please refer to our previous studies [33, 34]. DNA content was determined according to our previous study [34].

### Microscopy observation

Mycelia of *T. reesei* RUT-C30 and ZC121 were placed onto a glass slide, covered with a cover glass, and observed with an inverted confocal laser scanning microscope SP8 (Leica, Germany) equipped with a 100 × 1.4 NA oil-immersion objective. The excitation wavelength used was 405 nm and the emission was checked in the range of 415–495 nm.

### Sporulation assay

*Trichoderma reesei* was cultivated on PDA plates at 28 °C for 5 days to obtain mature mycelia. A portion of the mature mycelia was inoculated onto another PDA plate and incubated at 28 °C for 5 days. The spores were then collected using 4 mL of sterilized 0.02% Tween 80. The spores were counted using a hemocytometer and light microscope.

### RNA-seq analysis

The total RNA of *T. reesei* RUT-C30 was extracted with the RNA extraction Kit (Omega Bio-tek, Germany). The purity, concentration and integrity of the isolated RNA was assayed by the NanoDrop^®^ spectrophotometer (Thermo Fisher, USA), the Qubit^®^ RNA assay kit on Qubit^®^ 3.0 Fluorometer (Life Technologies, USA) and the RNA Nano 6000 assay kit with the Bioanalyzer 2100 system (Agilent Technologies, USA), respectively.

Illumina RNA sequencing was carried out with two duplicates by Genscript following their standard analysis method. Gene FPKMs were computed by summing the FPKMs of the transcripts in each gene group with Cuffdiff (v1.3.0). FPKM represents “fragments per kilobase of exon per million fragments mapped,” which is calculated from the length of the fragments and the reads count mapped to each fragment. Genes with the absolute value of log_2_ (fold change) ≥ 1 and the corrected *p* values less than 0.05 were assigned as significantly differentially expressed genes (DEGs) by Cuffdiff (v2.2.1). GO enrichment analysis of DEGs was implemented with Perl module (GO::TermFinder) [35]. GO terms with corrected *p* value less than 0.05 were significantly enriched among the differentially expressed genes. R functions (phyper and *q* value) were used to test for the statistical enrichment of the differentially expressed genes among the KEGG pathways. KEGG pathways with corrected *p* value less than 0.05 were considered to be significantly enriched among the differentially expressed genes.

## Results

### *Trichoderma reesei* recombinant strains with off-target mutagenesis displayed markedly reduced cellulase production, but significantly enhanced sorbicillinoids production

**Fig. 1:**
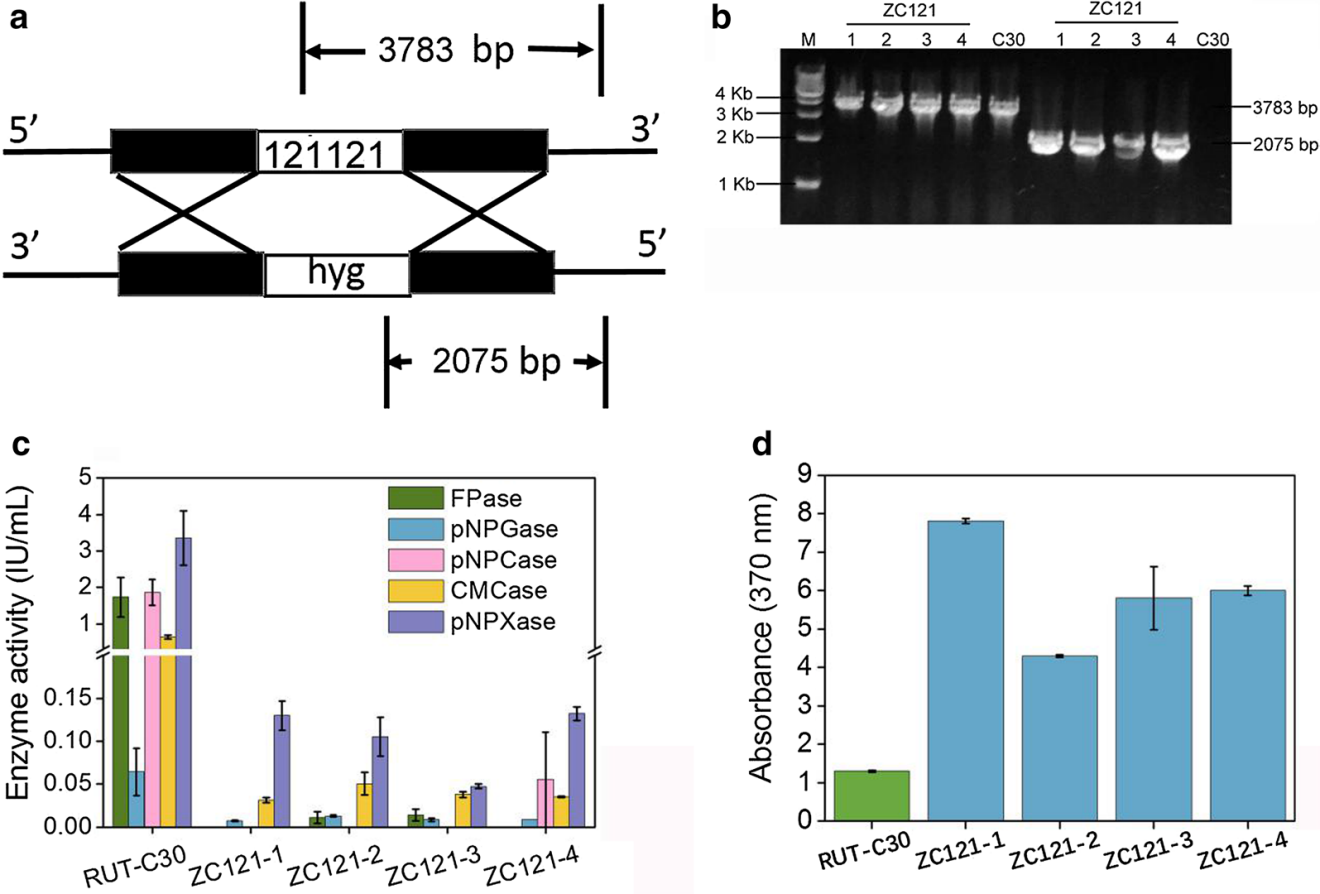
**a** Schematic description of the knockout of gene 121121 by homologous recombination using plasmid pXBthg-121121 (Additional file 1: Fig. S1). **b** PCR cloning of the gene fragments 3783 bp and 2075 bp as shown in **a** from *T. reesei* RUT-C30 and the recombinant strains ZC121-1, ZC121-2, ZC121-3, and ZC121-4. **c** Cellulase and hemicellulase activities in the culture supernatant of *T. reesei* RUT-C30, ZC121-1, ZC121-2, ZC121-3, and ZC121-4 grown on 2% cellulose were measured on day 5, including the activities of FPase (the filter paper activity), pNPGase (the BGL activity), pNPCase (the CBH activity), CMCase (the CMC activity), and pNPXase (the β-xylosidase activity). Meanwhile, **d** the corresponding yellow pigment production was determined by measuring the absorbance at 370 nm. The error bars indicate the standard deviations of three biological replicates

Gene 121121 encodes a candidate fungal regulatory protein that contains a Zn(2)Cys(6) fungal-type DNA binding domain. Gene 121121 is located next to β-glucosidase gene, *cel3d* [46]. Both genes belong to a tightly co-expressed genome region as identified by analysis of transcriptional data of *T. reesei* producing cellulases [46]. Moreover, gene *cel3d* was considered to be significantly involved in cellulase production by *T. reesei* [33]. Based on these previous findings, we presumed that gene 121121 might play a role in *T. reese*i cellulase production. Gene 121121 did not affect cellulase production when cells were grown on lactose [46], although no study was found which explored the effect of deletion of gene 121121 on cellulase synthesis. Therefore, we attempted to knockout gene 121121 in *T. reesei* RUT-C30 using homologous recombination mediated by AMT (Fig. 1a and Additional file 1: Fig. S1).

Four transformants, ZC121-1, -2, -3, -4, were obtained after selection using the marker, hygromycin. Unfortunately, the deletion of 121121 was not successful as shown by PCR result that gene 121121 was successfully cloned from all of four transformants, individually (Fig. 1b). This unsuccessful deletion was further confirmed by whole genome resequencing using NGS sequencing (Additional file 1: Fig. S4). However, we have successfully cloned the gene, hyg, in the four transformants mentioned above, demonstrating that the knockout cassette was randomly inserted into the chromosome of these strains by off-target mutagenesis [36].

Cellulase production in the four recombinant strains induced by cellulose on day 5 was assayed as described (Fig. 1c). The FPase, pNPCase, CMCase, pNPGase, and pNPXase activities of all four mutants were in the range of 0–1.9 IU/mL, 0.007–0.21 IU/mL, 0–0.37 IU/mL, 0.03–1.2 IU/mL, and 0.1–2.9 IU/mL, respectively, showing noticeably reduced cellulase activities when compared to that of RUT-C30, which was 6.1 IU/mL FPase, 0.5 IU/mL pNPCase, 6.8 IU/mL CMCase, 2.1 IU/mL pNPGase, and 4.6 IU/mL pNPXase. Obviously, the off-target mutagenesis caused serious inhibition of cellulase and hemicellulase production in *T. reesei*.

Surprisingly, we observed that these mutants displayed a much greater yellow color production than RUT-C30 both in liquid culture (Additional file 1: Fig. S4) and on the PDA plates (Additional file 1: Fig. S5), indicating far more sorbicillinoids were produced. For quantitative comparison, the absorbance of liquid cultures of *T. reesei* at 370 nm was used to measure the sorbicillinoids’ production (Fig. 1d) on cellulose [31]. The sorbicillinoids’ production in strains ZC121-1, ZC121-2, ZC121-3, and ZC121-4 was increased by 6, 3.3, 4.5, 4.6-fold, respectively, when compared to RUT-C30 production. In contrast to the dramatic decrease of cellulase and hemicellulase activities on cellulose, a significantly enhancement of yellow pigment production was observed by these recombinant strains. This finding demonstrated that off-target mutagenesis in strain ZC121 has switched cellulase production to sorbicillinoids’ production on cellulose. The recombinant strain ZC121-1 was selected for further study and was referred to as ZC121 because it displayed the highest yellow pigment production found in this study.

### Hyperproduction of sorbicillinoids in strain ZC121 is a constitutive process

**Fig. 2:**
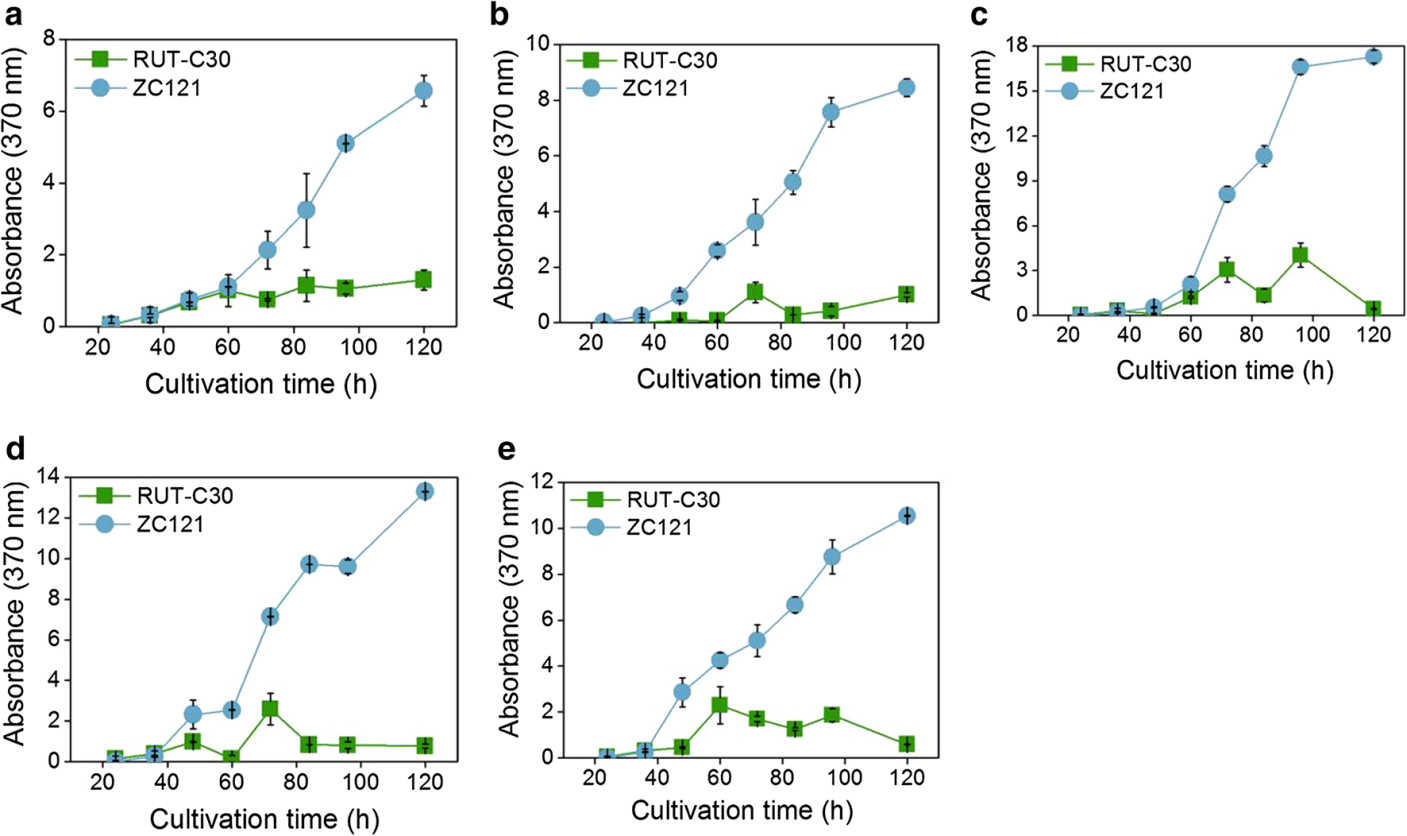
The sorbicillinoids’ production in the culture supernatant of *T. reesei* RUT-C30 and ZC121 grown on TMM medium containing **a** cellulose, **b** lactose, **c** glucose, **d** galactose and **e** glycerol, respectively, was assayed by measuring the absorbance at 370 nm at the indicated time points. Error bars indicate SDs from three independently grown cultures

In previous studies, the ability of *T. reesei* to produce sorbicillinoids was reported to vary with different carbon sources [19, 37–39]. To see whether the superior sorbicillinoids’ production of strain ZC121 compared to strain RUT-C30 is dependent on carbon source, both the sorbicillinoids and cellulase production of strains ZC121 were measured during the time course of growth on TMM containing cellulose, lactose, glucose, galactose or glycerol as the individual carbon source (Fig. 2 and Additional file 1: Fig. S6). The maximal absorbance at 370 nm of the culture supernatant of strain ZC121 was 6.6, 8.4, 17.3, 13.3 and 10.5 for cellulose, lactose, glucose, galactose and glycerol, respectively. Note that these values are 5.1, 8.4, 4.3, 5.1 and 4.5-fold that of RUT-C30 (Fig. 2). Obviously, strain ZC121 produced remarkably increased sorbicillinoids on all tested carbon sources. The highest sorbicillinoids’ production was found with glucose as the carbon source at 120 h of growth, followed by galactose, glycerol, lactose, and cellulose—in descending order. An absorbance of OD_370_ = 17.3 for the culture supernatant from growth on glucose corresponds to a concentration of 627 μg/mL yellow pigments (see “Materials and methods” section). Except for growth on cellulose, both ZC121 and RUT-C30 display very low cellulase production on lactose, glucose, galactose, and glycerol (Additional file 1: Fig. S5), which is reasonable because these four carbon sources have been shown to be inefficient inducers cellulases.

**Fig. 3:**
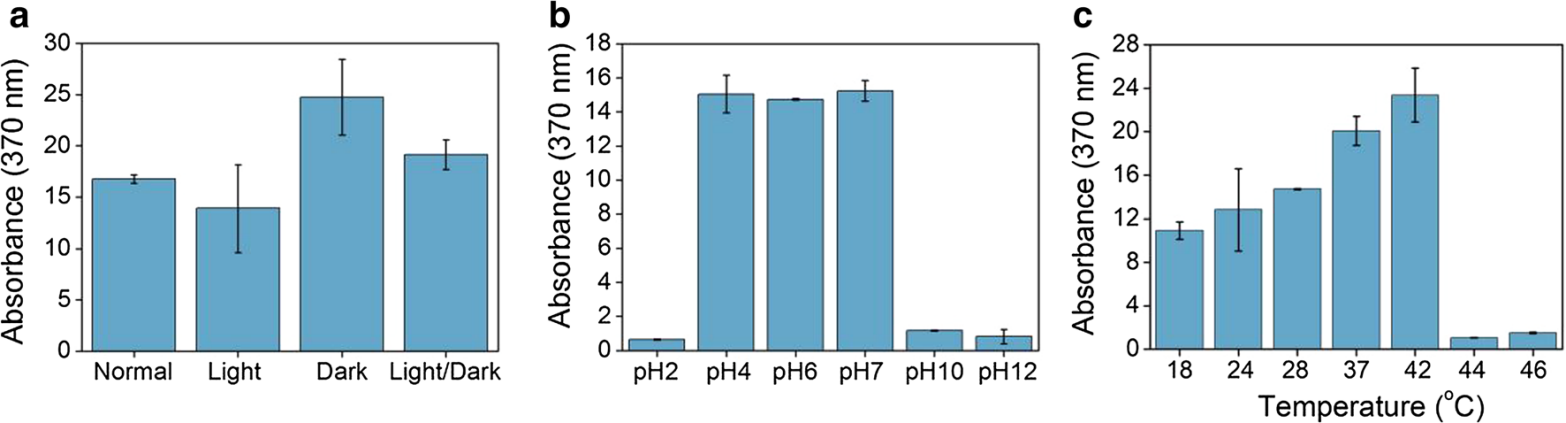
The effect of light (**a**), pH (**b**) and temperature (**c**) on the sorbicillinoids’ production ability of *T. reesei* ZC121. Unless otherwise indicated, *T. reesei* was cultivated on TMM + glucose (pH 6) at 28 °C with 200 rpm for 5 days. Error bars indicate SDs from three independently grown cultures

Using TMM + glucose as the culture medium, we also tested the effects of other culture conditions on the sorbicillinoids’ production ability of ZC121, including light, pH and temperature. Strain ZC121 was grown under normal lab light condition, constant light, constant darkness, or cycles of 12 h light–12 h dark (light–dark cycles) for 120 h. The highest absorbance (OD_370_ = 24.7) was observed for cell growth under constant darkness, whereas the absorbance under the other three light conditions were comparable internally (Fig. 3). It seems that the lighting condition only affects the production of sorbicillinoids in strain ZC121 to some limited extent, with the highest sorbicillinoids’ production observed for growth under constant darkness (Fig. 3a). However, strain ZC121 displayed excellent sorbicillinoids’ production ability under all tested light conditions. Strain ZC121 exhibited the hyperproduction of yellow pigment at pH 4, 6 and 7, but displayed sharply reduced pigment production at extreme pH values (pH 2, 10 and 12), which was probably related to the poor growth of strain ZC121 under these extreme pH conditions (Fig. 3b). The sorbicillinoids’ production declined somewhat when the culture temperature was decreased to 18 °C, but growth at 18 °C and 24 °C still maintained 74% and 87% of growth at 28 °C, respectively (Fig. 3c). In contrast, yellow pigment production was enhanced along with the increased temperature up to 42 °C (Fig. 3c). Beyond 42 °C, the sorbicillinoids’ production was reduced sharply, because strain ZC121 did not grown at all (Fig. 3c). Obviously, the high production performance of ZC121 was maintained over a limited range as the culture temperature was varied from the optimum temperature of 28 °C. In summary, the hyperproduction of sorbicillinoids in *T. reesei* ZC121 was a constitutive process, independent of carbon source, light, pH, and temperature.

### Identification of the off-target mutagenesis in recombinant strain ZC121

The random insertion of the knockout cassette of pxBthg-121121 (Additional file 1: Fig. S2) into the chromosome of *T. reesei* ZC121 could cause collateral mutations, which might contribute to the outstanding yellow pigment production shown by ZC121. Therefore, the identification of the insertional site might help us further understand the regulatory mechanism of sorbicillinoids’ biosynthesis. To this end, the whole genome resequencing using NGS sequencing was employed to find the random insertional sites. The whole genome resequencing of *T. reesei* ZC121 resulted in a total of 16,078,934 150-bp paired-end reads with mean depth coverage of 89.78% and Q30 percentage of 92.42%. These clean reads covered 89.78% of the reference genome of RUT-C30. The NGS sequencing result (NCBI Accession Number: SRR6906202) show that one copy of the cassette was probably inserted at 1–60 bp of *T. reesei* RUT-C30 genome KI911238.1 (https://www.ncbi.nlm.nih.gov/nuccore/572281258/). The insertion site was at the telomere of chromosome IV of *T. reesei*. No coding genes were found in the neighboring sequence of 3 kb.

### Identification of sorbicillinoids in the culture supernatant of strain ZC121

To determine which types of sorbicillinoids were produced by strain ZC121, LC–MS analysis of the culture supernatant of strain ZC121 cultivated on glucose for 5 days was performed (Table 1). A total of seven known sorbicillinoid-related compounds were identified, including sorbicillin, sorbicillinol, bisorbicillinol, dihydrosorbicillinol, oxosorbicillinol, bisvertinolone, and dihydrobisvertinolone. Furthermore, five structure-unknown compounds were detected. These compounds have been reported to be sorbicillinoid-related but are not named [19]. Peak area was utilized to roughly assess the abundance of these compounds (Table 1). Sorbicillinol was the most abundant product; bivertinolone was second. Sorbicillinol has been found to be the major sorbicillinoid-related product in several studies [13, 19, 25] and considered to be the building block for the other sorbicillinoids [19]. Bivertinolone can inhibit the biosynthesis of β-1,6-glucan and is an effective 1,1-diphenyl-2-picrylhydrazyl (DPPH) radical scavenger [40]. Its direct precursor, oxosorbicillinol, was also abundantly accumulated in the supernatant of strain ZC121. Only a trace amount of sorbicillin was detected, which agreed with the early findings [41].

**Table 1:** Metabolite profiling for sorbicillinoids in the culture broth of strain ZC121 grown on glucose for 5 days

Compound	Name	Formula	Acquired [M+H]^+^	RT (min)	Peak area
1	Sorbicillinol	C_14_H_16_O_4_	249.1135	6.94	333,191
2	Bisvertinolone	C_28_H_32_O_9_	513.2125	12.08	300,654
3	Oxosorbicillinol	C_14_H_16_O_5_	265.108	9.03	111,253
4	Bisorbicillinol	C_28_H_32_O_8_	497.2175	11.89	99,103
5	Dihydrobisvertinolone	C_28_H_34_O_9_	515.228	12.57	40,780
6	Dihydrosorbicillinol	C_14_H_17_O_4_	251.13	7.89	22,754
7	Sorbicillin	C_14_H_16_O_3_	233.1178	5.63	7002
8	Unknown	C_12_H_17_ON	192.1388	5.26	36,046
9	Unknown	C_11_H_12_O_3_	193.0865	6.67	35,210
10	Unknown	C_15_H_20_O_4_N2	293.1501	4.56	9430
11	Unknown	C_12_H_14_O_3_	207.1021	6.94	5678
12	Unknown	C_15_H_20_O_5_N_2_	309.145	4.58	1021

### Characterization of *T. reesei* ZC121

**Fig. 4:**
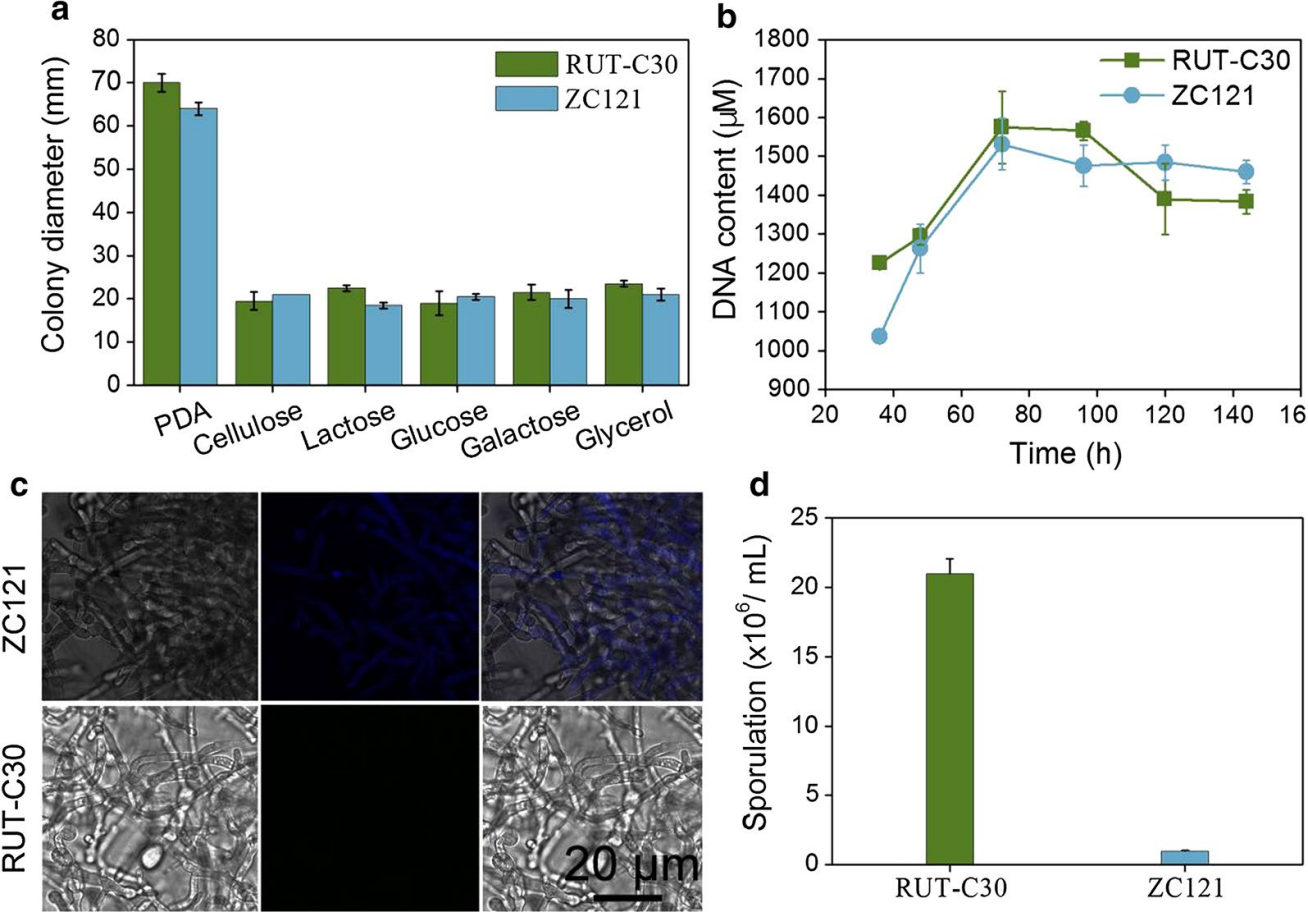
Characterization of *T. reesei* ZC121. Diameters of the colonies of *T. reesei* RUT-C30 and ZC121 grown on PDA plates, and TMM plates with 2% cellulose, 2% lactose, 2% glucose, 2% galactose and 2% glycerol, respectively, were measured (**a**). Meanwhile, the growth of RUT-C30 and ZC121 cultured in TMM + 2% cellulose for cellulase production was determined by measuring the cellular DNA content (**b**), while their hyphae were profiled by fluorescence microscopy at Ex/Em = 405/415–495 nm (**c**). The sporulation of *T. reesei* RUT-C30 and ZC121 on PDA plates was counted on day 5 (**d**)

Growth of strains ZC121 and RUT-C30 was studied by measuring colony diameters on PDA plates and TMM plates with different carbon sources (i.e., cellulose, lactose, glucose, galactose or glycerol) (Fig. 4a and Additional file 1: Fig. S6). The colony diameters of these two strains were nearly the same regardless the carbon sources (Fig. 4a). Furthermore, the growth of strain ZC121 and RUT-C30 in TMM + 2% cellulose was assayed by DNA content measurement (Fig. 4b), as we were unable to accurately measure the dry *T. reesei* biomass due to the interference from insoluble cellulose. No significant difference of growth was observed between strain ZC121 and RUT-C30 (Fig. 4b). These results suggest that off-target mutagenesis did not affect the growth of *T. reesei* on different carbon sources.

The morphology of ZC121 was different from that of RUT-C30. The number of the branched hyphae of ZC121 decreased and its hyphae became thinner and longer when compared to RUT-C30 (Fig. 4c). Also, the sorbicillinoids were not only secreted out of the cells, but also filled the fungal cell resulting in blue fluorescence emission at 405 nm excitation—as observed under fluorescence confocal microscopy (Fig. 4c). Furthermore, the spore amount of strain ZC121 was 1 × 10^6^/mL, only 4.8% of that of RUT-C30 (2.1 × 10^7^/mL) (Fig. 4d), demonstrating that the off-target mutagenesis leads to noticeable reduction of sporulation in *T. reesei* ZC121. This negative link between secondary metabolite production and conidiation in fungi was frequently found in early studies [20]. For example, when deleting the velvet protein VeA in *A. nidulans*, the abolishment of penicillin and aflatoxin secondary metabolite production was accompanied with the enhancement in asexual conidiation. The low level expression of sorbicillinoid-related genes during asexual growth was also reported [12]. Overall, with the off-target mutagenesis, the growth rate and biomass of *T. reesei* ZC121 was not affected significantly, but its morphology changed with marked sporulation reduction.

### Transcription patterns of strain ZC121

RNA-seq analysis was performed to understand how the off-target mutagenesis influences the transcriptional level of strain ZC121. The sequences of the total reads were mapped to the reference genome of *T. reesei* RUT-C30 (https://www.ncbi.nlm.nih.gov/genome/323?genomeassembly_id=49799) with coverage of 93.24–95.63%. A total of 9544 unique transcripts were detected. Genes were differentially expressed between the two strains when the average reads of the corresponding transcripts differed with |log2Ratio| ≥ 1 and adjusted *p* values ≤ 0.05. By comparing strain ZC121 to RUT-C30, we obtained 638 and 1006 differentially expressed genes (DEGs) under cellulose and glucose growth conditions, respectively (Additional file 1: Table S4). Among these, 341 DEGs were found under both glucose and cellulose growth conditions, of which 327 DEGs expression changing trends were identical between these two carbon sources, whereas 14 DEGs expression changing trends were opposite (Additional file 1: Table S4).

**Fig. 5:**
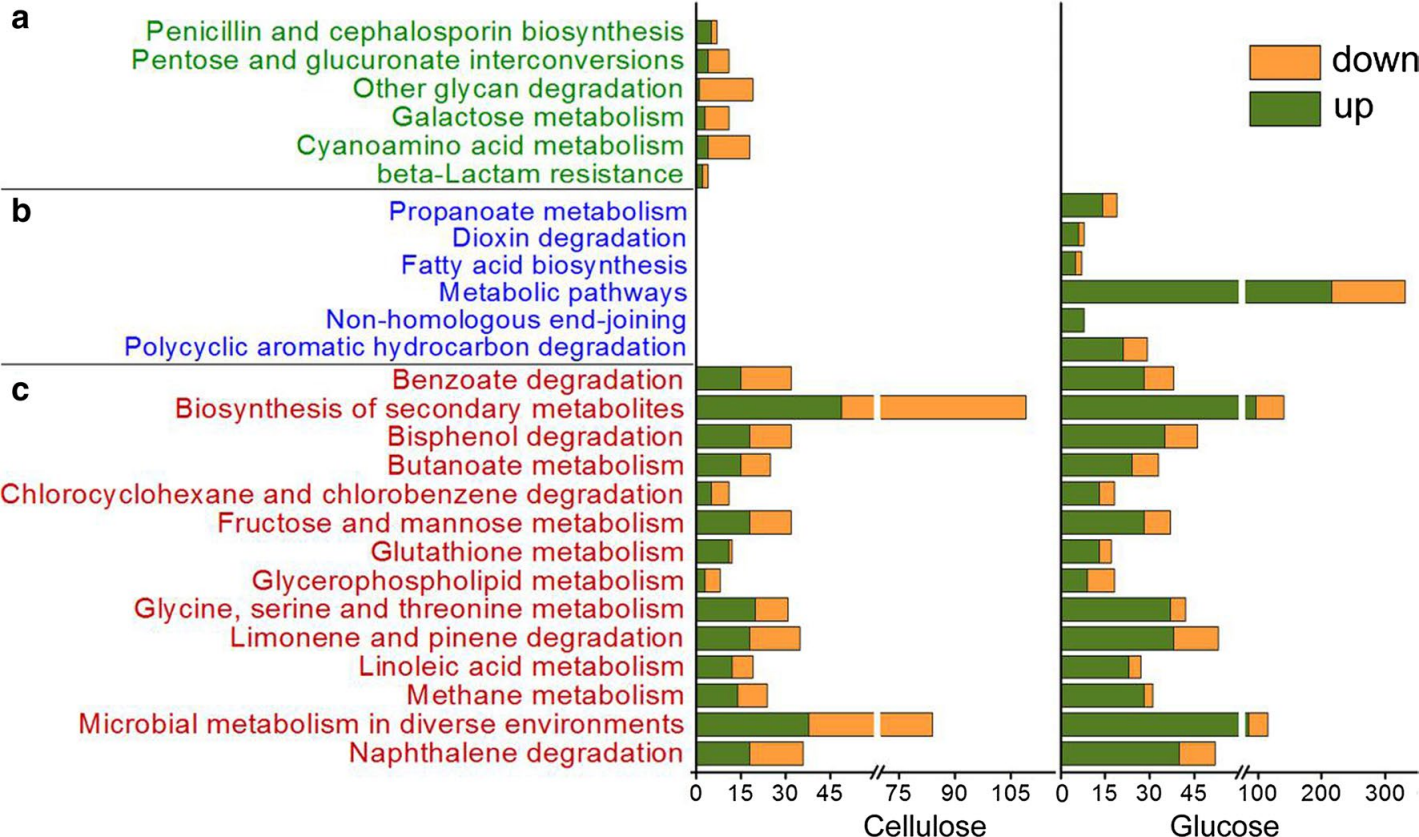
Kyoto Encyclopedia of Genes and Genomes (KEGG) enrichment analysis of DEGs in strain ZC121 in comparison with the parental strain RUT-C30 on cellulose or glucose. The y axis represents the name of the top 20 enriched pathways, and the x axis shows the number of DEGs in the enriched pathways. The enriched pathways were categorized into pathways enriched only under glucose condition (a), only under cellulose condition (b), and under both glucose and cellulose conditions (c)

KEGG pathway enrichment analysis showed that 14 out of the top 20 enriched pathways were shared by growth on both glucose and cellulose conditions (Fig. 5c). This result implies that the impact of the off-target mutagenesis in ZC121 at the transcription level share a lot in common between growth on different carbon sources. The “other glycan degradation” was enriched only on cellulose (Fig. 5a). Most genes in this pathway were related to cellulase production induced by cellulose and were down-regulated, notably. This result is in line with reports of the inhibited cellulase production in strain ZC121 for growth on cellulose in comparison with RUT-C30.

**Fig. 6:**
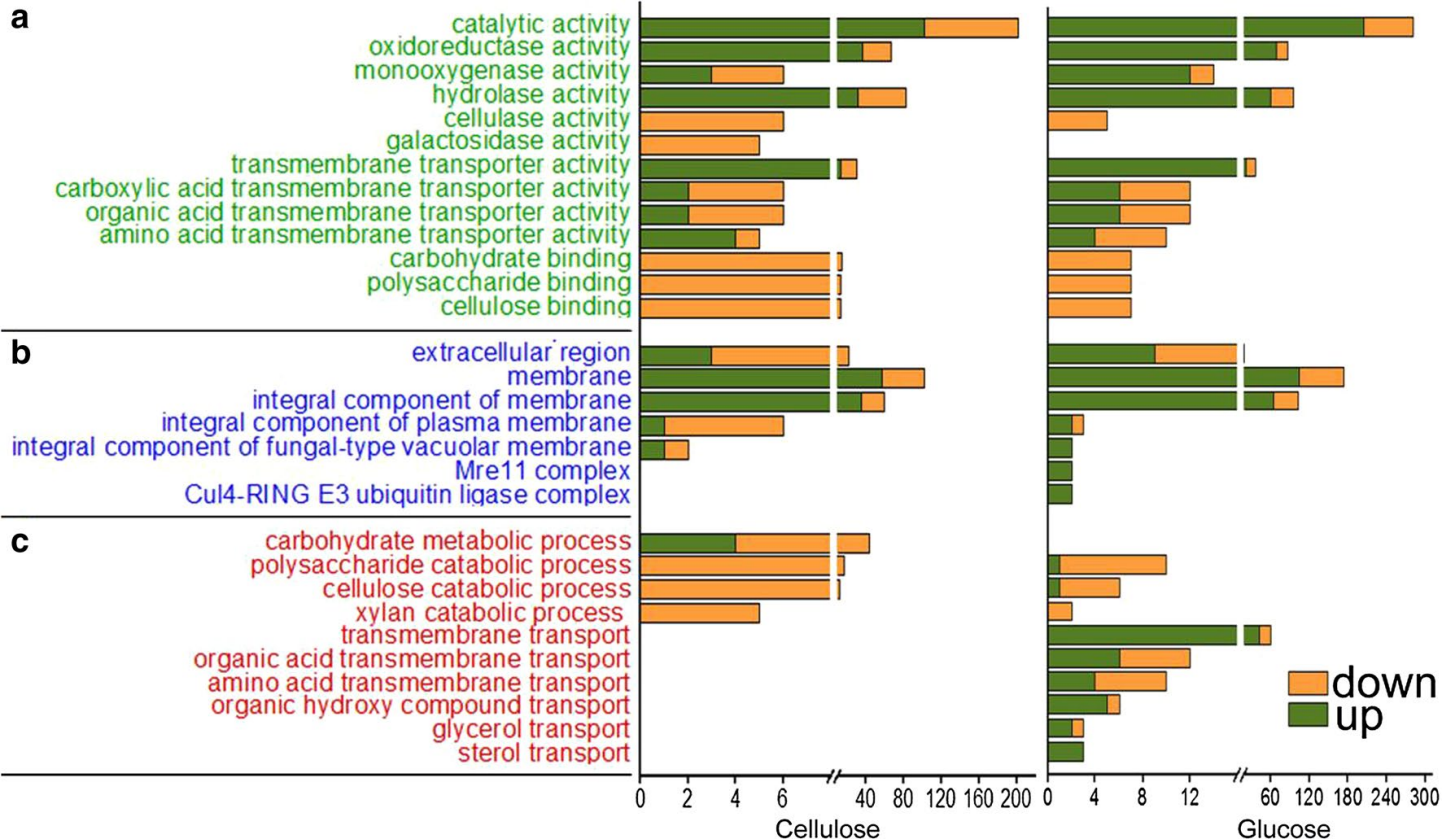
Gene ontology (GO) functional enrichment analysis of DEGs of strain ZC121 compared to the parental strain RUT-C30 grown on cellulose and glucose as the sole carbon source, respectively. The y axis represents the name of the most enriched GOs that belong to different ontologies: (a) the molecular function, (b) the cellular component and (c) the biological process, while the x axis represents the number of DEGs in each enriched GO

The enriched molecular functions “catalytic activity” and “cellulose binding” were observed for growth on both glucose and cellulose (Fig. 6a), including two enriched subcategories “oxidoreductase activity” and “hydrolase activity, acting on glycosyl bonds”. “Transmembrane transporter activity” was enriched only under glucose growth conditions (Fig. 6a).

For the enriched cellular components, both “membrane” and “extracellular region” were enriched in the presence of glucose or cellulose (Fig. 6b), which is reasonable given that both cellulases and sorbicillinoids are secreted outside of the fungal cells. Under the “membrane” category, DEGs are mainly involved in the intrinsic component of membrane (Fig. 6b), including the plasma membrane, vacuolar membrane, nuclear membrane, and mitochondrial membrane. Deletion of gene 121121 might pose a significant change on cell structure of *T. reesei*, which might be responsible for the morphology change mentioned above (Fig. 4c); as well as the condition defect (Fig. 4d).

In the presence of cellulose, the most enriched DEGs in the category of biological process belong to “carbohydrate metabolic process” which mainly included “xylan catabolic process” and “cellulose catabolic process” (Fig. 6c). Most of these DEGs were downregulated, which is consistent with the markedly reduced cellulase production and the result of the KEGG pathway enrichment analysis. Under glucose growth conditions, the most enriched biological process changed to “transmembrane transport” and “oxidation–reduction process” (Fig. 6c).

### Most DEGs involved in the cellulase production were downregulated in *T. reesei* ZC121 on cellulose

There are 74 genes known or predicted to be related to cellulase/hemicellulase production in *T. reesei*, of which 43 were DEGs with notably downregulated mRNA levels, except gene *xdh1* (Additional file 1: Table S5). Specifically, the transcriptional levels of cellulase/hemicellulase genes, such as cellobiohydrolase *cel6a* (CBH II), endoglucanases *egl1, egl2, egl3, egl4,* and *egl5*, β-glucosidases *cel3a* (*bgl1*), *cel3d*, *cel3e*, *cel1a*, and *cel1b*, β-xylosidase *bxl1,* and xylanases *xyn1*, *xyn2* and *xyn3* were all reduced markedly in strain ZC121 (Table 2), matching well with the nearly abolished cellulase/hemicellulase activities found in the supernatant of ZC121 culture. The auxiliary proteins, such as swollenin (encoded by *swo1*) [42], the glycoside hydrolase Family 61 (GH61 s) GH61b and GH61a [43] and the cellulose-induced proteins CIP-1 and CIP-2 [44], which have been shown to improve the hydrolysis of cellulose, were also downregulated significantly in ZC121 grown on cellulose. In addition, three well-known cellulase transcription activators *xyr1* [45], *ace3* [46], and *crt1* [47] were significantly downregulated in strain ZC121 grown on cellulose, whereas cellulase transcription repressor, *cre1* [48], was downregulated under both cellulose and glucose growth conditions.

**Table 2:** The main DEGs related to (hemi) cellulase in strain ZC121 (C121)

Gene ID^a^	Gene name	Description	log2 (C121/CC30)	*p* value	Category
122470	*cel6a*	Exoglucanase 2	− 6.43	5.00E−05	Cellulase
5304	*egl1*	Endoglucanase I precursor	− 5.75	5.00E−05	Cellulase
124931	*egl2*	Endoxylanase II	− 2.63	0.0001	Cellulase
72489	*egl3*	Endoglucanase III	− 6.74	5.00E−05	Cellulase
139633	*egl4*	Endoglucanase-4	− 5.70	5.00E−05	Cellulase
25940	*egl5*	Endo-1,4-beta-glucanase V	− 2.50	5.00E−05	Cellulase
136547	*cel3a*	Beta-d-glucoside glucohydrolase I	− 4.35	5.00E−05	Cellulase
122639	*cel3d*	Hypothetical protein	− 1.94	5.00E−05	Cellulase
74305	*cel3e*	Hypothetical protein	− 1.88	5.00E−05	Cellulase
127115	*cel1a*	Beta-glucosidase	− 3.13	5.00E−05	Cellulase
77989	*cel1b*	Glycoside hydrolase	− 2.90	5.00E−05	Cellulase
122518	*cel61b*	Endoglucanase VII	− 6.84	5.00E−05	Cellulase
77521	*bxl1*	Family 43 glycoside hydrolase	− 1.32	0.0017	TF
38418	*xyn1*	Endo-1,4-β-xylanase 1	− 1.45	0.0009	Hemicellulases
124931	*xyn2*	Endo-1,4-β-xylanase 2	− 2.63	0.0001	Hemicellulases
23616	*xyn3*	Xylanase III	− 6.17	5.00E−05	Hemicellulases
104220	*swo1*	Swollenin	− 3.52	5.00E−05	Nonenzymatic cellulose attacking enzymes
121449	*cip1*	Hypothetical protein	− 6.30	5.00E−05	Nonenzymatic cellulose attacking enzymes
125575	*cip2*	Hypothetical protein	− 5.19	5.00E−05	Nonenzymatic cellulose attacking enzymes
98788	*xyr1*	Xylanase regulator 1	− 2.28	5.00E−05	TF
98455	*ace3*	Hypothetical protein	− 2.29	5.00E−05	TF
109243	*crt1*	General substrate transporter	− 3.04	5.00E−05	TF
23706	*cre1*	Hypothetical protein	− 1.63	0.0001	TF

^a^Gene ID was assigned based on the *T. reesei* RUT-C30 genome database (https://www.ncbi.nlm.nih.gov/genome/323?genome_assembly_id=49799)

### DEGs involved in sorbicillinoid biosynthesis and other secondary metabolism

**Fig. 7:**
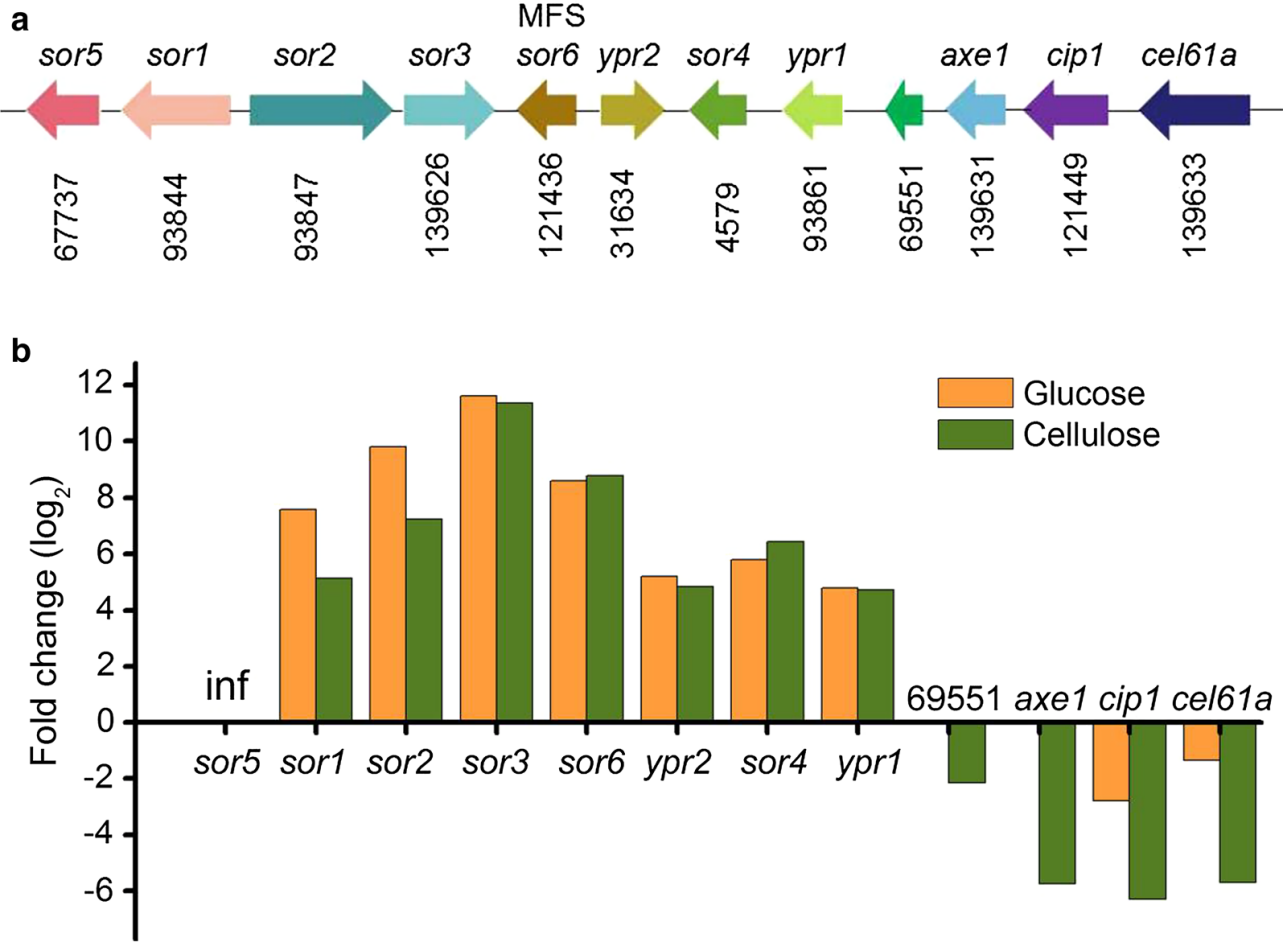
**a** Genomic organization of the sorbicillinoid gene cluster and its neighboring cellulase-related genes in *T. reesei*. The relative transcription levels of these genes in strain ZC121 to RUT-C30 are shown under both cellulose and glucose conditions **b**. Gene 102500 and *axe1* were not DEGs under glucose condition, so their fold change was not presented. Inf: the transcript of *sor5* was not detected in RUT-C30, but in ZC121

Genes involved in the biosynthesis of sorbicillinoids were clustered in the *T. reesei* genome and named the “sorbicillinoid gene cluster” [14, 25]. This *T. reesei* gene cluster contains a highly reducing PKS Sor 1, a non-reducing PKS Sor 2, a flavin adenine dinucleotide (FAD)-dependent monooxygenase Sor3, a FAD/flavin mononucleotide-containing dehydrogenase Sor4, two transcription factors YPR1 and YPR2 [3], a MFS transporter Sor6, and a short-chain dehydrogenase/reductase Sor 5 (Fig. 7 and Additional file 1: Table S2). We provisionally named genes in the sorbicillinoid gene cluster in this study, because the naming of this cluster was inconsistent in previous studies [25, 26]. It is worth noting that all genes in the sorbicillinoid cluster were upregulated significantly in strain ZC121 when using glucose or cellulose as the sole carbon source (Fig. 7b and Additional file 1: Table S2). This result agrees well with the excellent overproduction of sorbicillinoids in strain ZC121 (Fig. 2). Under both conditions tested, the expression of Sor5 was detected in strain ZC121, but not in RUT-C30 (Fig. 7b and Additional file 1: Table S2). Gene *sor5* in this study, which is designated as *sor7* in the literature, encodes a short-chain dehydrogenase/reductase [14]. Its ortholog from *P. rubens* was not located in the sorbicillinoid gene cluster. Moreover, gene *sor5* was not found in most other fungi. It seems that the off-target mutagenesis activates the expression of gene *sor5*, which might play a role in the notable overexpression of genes in the sorbicillinoid cluster. However, how the activation of gene *Sor5* functions in *T. reesei* ZC121 is unclear, because studies performed on gene *sor5* are rare.

In the previous study, an extensive search for PKS-, NRPS- and hybrid synthetase genes using basic local alignment search tool (BLAST) revealed the 11 PKS-, 11 NRPS- and 4 hybrid synthetase genes in the *T. reesei* genome (http://genome.jgipsf.org/pages/blast.jsf?db=Trire2), which could be appointed to 23 distinct gene clusters [21]. Also, a total of 31 predicted transcription factor (TF) genes were found in a 50 kb radius of these synthase genes. Among them, five synthases and four TFs were significantly upregulated in strain ZC121 under both tested conditions (Additional file 1: Table S6), of which two synthases and two TFs (Additional file 1: Table S2) are involved in the sorbicillin biosynthesis pathway, as we discussed above. The transcription level of pks8 (90904) were increased under both conditions (Table 3). Its homologues, adaA from *A. niger* [10] or vrtA from *Penicillium aethiopicum* [11], were involved in anthracenone and naphthacenedione biosynthesis, respectively. A hybrid PKS-NRPS synthase (128011) and its neighboring TF (74475) was significantly upregulated (Table 3), whose closest annotated hit was fusaproliferin synthase [21]. Fusaproliferin, a mycotoxin from *Fusarium* spp. PKS 77957, is a nonribosomal peptide synthetase, whose homologue in *Aspergillus fumigatus* is essential for fumigaclavine C production. Fusaproliferin has been studied extensively [49]. This outcome shows that other secondary metabolites, other than sorbicillinoids, might also be produced in strain ZC121 and thus it is worth studying them in the future.

**Table 3:** DEGs that are synthetases and transcriptional factors involved in secondary metabolism, which were found under both cellulose and glucose conditions

Gene ID^a^	Gene name	Description	log2 (C121/CC30)		*p* value		Category
			Cellulose	Glucose	Cellulose	Glucose	
90904	90904	Putative TPA: polyketide synthase	4.98278	5.88729	5.00E−05	5.00E−05	Synthase
128011	128011	Hypothetical protein	3.33221	2.94805	5.00E−05	0.0001	TF
74475	74475	Hypothetical protein	2.96889	3.35194	5.00E−05	0.0115	TF
77957	77957	Putative nonribosomal peptide synthase GliP-like protein	4.56203	2.51002	5.00E−05	5.00E−05	Synthase
77229	77229	Hypothetical protein	2.27	2.09	5.00E−05	5.00E−05	TF

^a^Gene ID was assigned based on the *T. reesei* RUT-C30 genome database (https://www.ncbi.nlm.nih.gov/genome/323?genome_assembly_id=49799)

## Discussion

Sorbicillinoids have potential pharmaceutical value as antimicrobial, antivirus, and anticancer agents [50]. Moreover, they could be utilized as pigments and food colorants (yellow pigments) as well. Since they were first discovered in 1948 from *Penicillium notatum* [51], studies related to sorbicillinoids have focused on finding new compounds with similar structure [52], elucidating chemical structures and biological activities [16, 50], establishing the complex biosynthetic pathway [41] and developing chemical synthesis methods [53]. However, both strain engineering to increase sorbicillinoids’ production and the relevant regulatory mechanism is less studied. *T. reesei* is well-known for its prominent enzyme-secreted ability using cellulose as the efficient inducer and widely utilized in industry as a work horse for the production of both cellulase and heterologous recombinant proteins [54]. For the first time, an overwhelming switch from cellulase production to sorbicillinoids’ production using cellulose as carbon source was reported in recombinant *T. reesei* strain ZC121. Coincidentally, an expression shift from cellulase-related genes to genes in the sorbicillinoid gene cluster was found by transcriptional profiling with steep downregulation of 42 genes involved in cellulase production and significant upregulation of all genes in the sorbicillinoid cluster. This switch would enable *T. reesei* to contribute more energy and metabolites to sorbicillinoids’ production, considering that cellulase production is a heightened energy-efficient process. This shift, at the same time, would allow the easier separation of sorbicillinoids from a culture supernatant that contains only low titers of cellulases. More importantly, *T. reesei* was first engineered to directly degrade cellulose to produce other platform chemical compounds, rather than proteins. The routine strategy for cellulose-based chemical production is the combination of *T. reesei* for cellulose degradation and other model industrial microorganisms for desired compound production via co-culturing [29] and consolidated bioprocessing [55], achieving the degradation of cellulose to produce platform compounds, such as isobutanol [29] and ethanol [56]. These strategies using multiple microorganisms often require complicated and stringent operations, with the compatibility between two or more working microorganisms as another major challenge [55]. Therefore, it is of great value to have *T. reesei* mutants that can produce just the necessary amount of cellulases/hemicellulases to support the host cells in the production of valuable products in high yield.

In most studies related to sorbicillinoids, glucose is usually used as the carbon source with the highest sorbicillinoids’ production of OD_370_ = 13 reported in the literature [31], which is lower than OD_370_ = 17.3 of strain ZC121 in this study. Upon growth on cellulose, *T. reesei* cultures with the deletion of gene XPP1 can produce a small amount of yellow pigment with OD_370_ ≈ 0.32 [31], much less than the OD_370_ = 6.6 we observed in strain ZC121. Strain ZC121 generated the highest reported amounts of sorbicillinoids when grown on cellulose or glucose, as far as we know. Other carbon sources, such as lactose, glycerol, and galactose have been reported to be utilized by *T. reesei* to produce remarkable amounts of sorbicillinoids. Furthermore, the sorbicillinoids’ production in fungi has been reported to be generally impacted by culture conditions, such as carbon source [9, 37], light exposure time [39], temperature, and pH. By contrast, the recombinant strain ZC121 displayed hyperproduction of sorbicillinoids regardless the culture conditions, so long as *T. reesei* can grow well. This constitutive hyperproduction would benefit the future industry application of strain ZC121 for sorbicillinoids’ production with great flexibility and easy fermentation operation.

Currently, sorbicillinoids are not produced by industry. Various efforts have been made to access these compounds by chemical synthesis in the laboratory and only for research purposes [41]. However, most of the chemical synthesis routes are cumbersome, as the structure of sorbicillinoids is complex [53]. Whether or not these chemical methods are applicable in the industry is still unknown. Moreover, the chemical method is less environment-friendly than the biosynthesis. Therefore, it is highly desired to have recombinant microorganisms that can produce constantly sorbicillinoids with high yield.

The global regulator *laeA* is well-known for being involved in the secondary metabolism by regulating some polyketides [57]. Protein LaeA directly interacts in the nucleus with transcription factors of the trimeric Velvet complex, consisting of the Velvet domain proteins VeA and VelB [57]. The heterotrimeric velvet complex VelB/VeA/LaeA correlates fungal development and secondary metabolism in response to light. Moreover, it is found that both the knockout and overexpression of gene *laeA* affected the expression of some sor genes [14]. Nevertheless, the mRNA level of all these three genes remained unchanged in strain ZC121 in comparison with RUT-C30. Moreover, VosA, a recently identified regulator of fungal sporogenesis [58], another binding partner of VelB [57], was also not affected in strain ZC121. This finding is in line with the observation that *T. reesei* ZC121 exhibited high sorbicillinoids’ production as a function of light exposure time as we showed above.

The major types of sorbicillinoids generated in strain ZC121 from growth on glucose were sorbicillinol and bisvertinolone as analyzed by LC–MS (Table 1). It is worth noting that bisvertinolone is a potential anticancer agent (acts by inhibiting β-1,6-glucan biosynthesis or serving as an effective 1,1-diphenyl-2-picrylhydrazyl (DPPH) radical scavenger [40]. Currently, it is only available by intricate chemical methods [53]. Bisvertinolone has never been reported to be the major product in *T. reese*i. Furthermore, the precursor of bisvertinolone, oxosorbicillinol was the third most abundant compound in the culture medium (Table 1). Thus, it seems that *T. reesei* ZC121 might serve as a good starting strain to obtain mutants that primarily produce bisvertinolone by further strain optimization.

Interestingly, four genes related to cellulase production are in close vicinity to the sorbicillinoid gene cluster, including gene 102500, *axe1*, *cip1* and *cel61a,* forming a “sorbicillinoid-cellulase” supercluster (Fig. 7a). In contrast to the remarkably upregulated expression of genes in the sorbicillinoid gene cluster, these four genes were all DEGs steeply downregulated in ZC121 on cellulose growth, while only two genes were DEGs markedly reduced on glucose growth (Fig. 7b). Genes encoding candidate carbohydrate-active enzymes (CAZy) for polysaccharide degradation are prone to clustering in the *T. reesei* genome, leading to 25 genomic regions of high CAZy gene density [59]. These regions usually possess genes participating in secondary metabolism as well, forming “superclusters”. These kinds of superclusters have also been observed for other secondary metabolites in other fungi, playing a key role in the corresponding secondary metabolite production [60–62]. However, whether and how the “sorbicillinoid–cellulase” supercluster impacts the secondary metabolism for sorbicillinoids and cellulase is still unknown and is worth exploring in future studies.

We have initially aimed to specifically knockout gene 121121 in *T. reesei* RUT-C30 to study its function on cellulase production by AMT utilizing homologous recombination; however, this approach unfortunately failed. Continued work gave rise to the unexpected off-target mutagenesis at the telomere region of chromosome IV. The insertion of T-DNA into the genome of the recipient cell is random [63]. This randomness of T-DNA integration leads to inefficient targeting, resulting in off-target mutagenesis. It has been shown that T-DNA can insert into the telomeric region by non-homologous recombination (NHR) [64]. Therefore, although the similarity between the flanking 1500-bp regions of gene 121121 and the integration site at the telomere of chromosome IV of *T. reesei* is not high (40.4% and 39.0% for the upstream and downstream flanking regions, respectively), off-target integration was observed at the telomere of chromosome IV of *T. reesei*. The inefficient gene targeting by AMT has been a long-standing issue in plant and fungi [36]. Strategies like generation of double strand breaks at genomic positions of interest, downregulation of enzymes involved in NHEJ pathway, and concomitant translocation of the homing endonuclease I-SceI, have been explored to improve targeted integration of AMT [36]. Nevertheless, target mutagenesis can be embraced as a potent mutagenesis strategy for strain engineering as we showed here, together with the traditional methods using nitrosoguanidine (NTG) or UV irradiation. The insertion of T-DNA into the telomeric region induced gross chromosome rearrangement [64]. Chromosome remodeling plays an important role in cellulase production [33, 34, 65]. It is tempting to speculate that the off-target mutagenesis probably gave rise to the excellent sorbicillinoids’ production ability of strain ZC121, which came about by chromosome rearrangement and subsequently affected gene expression as we found using transcriptome analyses.

## Conclusion

We constructed recombinant *T. reesei* strain ZC121 from *T. reesei* RUT-C30 by off-target mutagenesis during the failed knockout of gene 121121. The knockout cassette for gene 121121 was found to insert at the telomere of chromosome IV of *T. reesei.* Strain ZC121 exhibited constitutive hyperproduction of sorbicillinoids under all tested culture conditions, including varied carbon source, light, pH, and temperature. Particularly, an overwhelming switch from cellulase production to sorbicillinoids production was observed in strain ZC121 on cellulose. Coincidently, a similar shift was also observed at the transcriptional level in ZC121 cultivated on cellulose, with steep downregulation of 42 genes involved in cellulase production and significant upregulation of all genes in the sorbicillinoid cluster. For the first time, *T. reesei* alone can degrade cellulose to directly produce valuable compounds (sorbicillinoids in this case) other than proteins, paving the way for the industrial production of cellulase-based chemical compounds.

## Additional file

**Additional file 1: Fig. S1.** Schematic illustration of the plasmid pXBthg-121121. hyg: homomycin resistance; LB, left border of binary vector; RB, right border of binary vector; Kan: kanamycin resistance; 121121-up: the 1500 bp upstream of gene 121121; 121121-down: the 1500 bp downstream of gene 121121. **Fig. S2.** The 1500-bp upstream and downstream regions of gene 121121. **Fig. S3.** The standard curve of sorbicillinoids. **Fig. S4.** The whole genome resequencing of strain ZC121 shows the unsuccessful deletion of gene 121121. **Fig. S5.** The color of the culture supernatant of *T. reesei* RUT-C30 (a) and ZC121 (b) grown on glucose. **Fig. S6.** Cellulolytic enzyme activities in the culture supernatant of *T. reesei* ZC121 and RUT-C30 grown on 2% cellulose, lactose, glucose, galactose and glycerol were assayed on day 5, including the activities of FPase (the filter paper activity) (a), pNPGase (the BGL activity) (b), pNPCase (the CBH activity) (c), CMCase (the CMC activity) (d) ahd pNPXase (the β-xylosidase activity) (e). The error bars indicate the standard deviations of three biological replicates. **Table S1.** Primers used in this study. **Table S2.** Comparative transcription levels of the sorbicillinoid gene cluster and its neighboring cellulase-related genes in *T. reesei* ZC121on cellulose and glucose.

## Abbreviations

pNPGase: the β-glucosidase activity
pNPCase: the CBH activity
CMCase: the CMC activity
FPase: the filter paper activity
pNPXase: the β-xylosidase activity
AMT: agrobacterium tumefaciens-mediated transformation
PDA: potato dextrose agar
SDB: sabouraud dextrose broth
TMM: Trichoderma minimal medium
MFS: major facilitator superfamily sugar transporter
PKSs: polyketides
NRPs: non-ribosomal peptides
DEGs: differentially expressed genes
GO: gene ontology
KEGG: Kyoto Encyclopedia of Genes and Genomes
NGS: next-generation sequencing
FPKMs: fragments per kilobase of transcript sequence per millions base pairs sequenced
RT-qPCR: quantitative real-time PCR
