# Supplementary material for: Constitutive hyperproduction of sorbicillinoids in Trichoderma reesei ZC121
Source: Biotechnol Biofuels. 2018 Oct 25;11:291. doi: 10.1186/s13068-018-1296-4 (PMC6202828; doi:10.1186/s13068-018-1296-4)
Supplement: Supplementary file 1 — Additional file 1: Fig. S1. Schematic illustration of the plasmid pXBthg-121121. hyg: homomycin resistance; LB, left border of binary vector; RB, right border of binary vector; Kan: kanamycin resistance; 121121-up: the 1500 bp upstream of gene 121121; 121121-down: the 1500 bp downstream of gene 121121. Fig. S2. The 1500-bp upstream and downstream regions of gene 121121. Fig. S3. The standard curve of sorbicillinoids. Fig. S4. The whole genome resequencing of strain ZC121 shows the unsuccessful deletion of gene 121121. Fig. S5. The color of the culture supernatant of T. reesei RUT-C30 (a) and ZC121 (b) grown on glucose. Fig. S6. Cellulolytic enzyme activities in the culture supernatant of T. reesei ZC121 and RUT-C30 grown on 2% cellulose, lactose, glucose, galactose and glycerol were assayed on day 5, including the activities of FPase (the filter paper activity) (a), pNPGase (the BGL activity) (b), pNPCase (the CBH activity) (c), CMCase (the CMC activity) (d) ahd pNPXase (the β-xylosidase activity) (e). The error bars indicate the standard deviations of three biological replicates. Table S1. Primers used in this study. Table S2. Comparative transcription levels of the sorbicillinoid gene cluster and its neighboring cellulase-related genes in T. reesei ZC121on cellulose and glucose. [file 13068_2018_1296_MOESM1_ESM.docx]

**Additional file 1:**


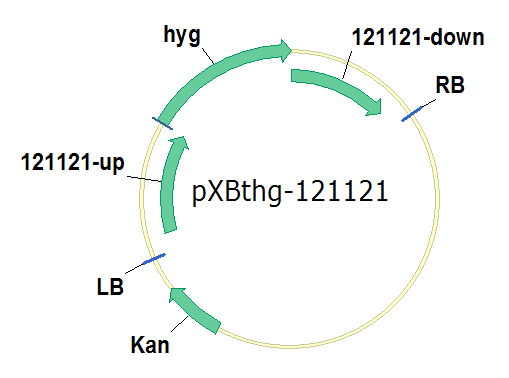


Fig. S1 Schematic illustration of the plasmid pXBthg-121121. hyg: [homomycin](file:///D:\Program%20Files%20(x86)\Youdao\Dict\7.5.0.0\resultui\dict\?keyword=homomycin) resistance; LB, left border of binary vector; RB, right border of binary vector; Kan: kanamycin resistance; 121121-up: the 1500 bp upstream of gene 121121; 121121-down: the 1500 bp downstream of gene 121121.


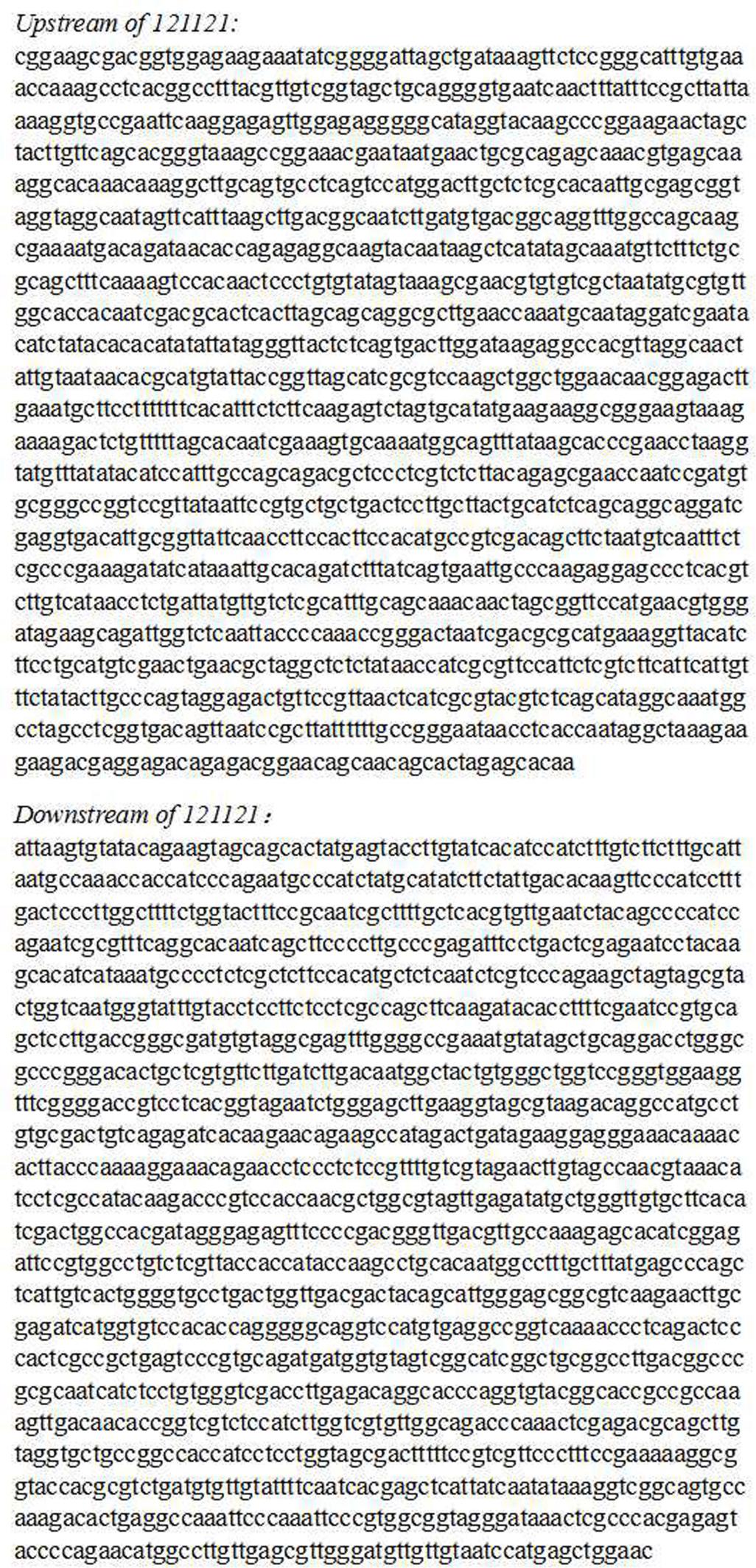


Fig. S2 The 1500-bp upstream and downstream regions of gene 121121


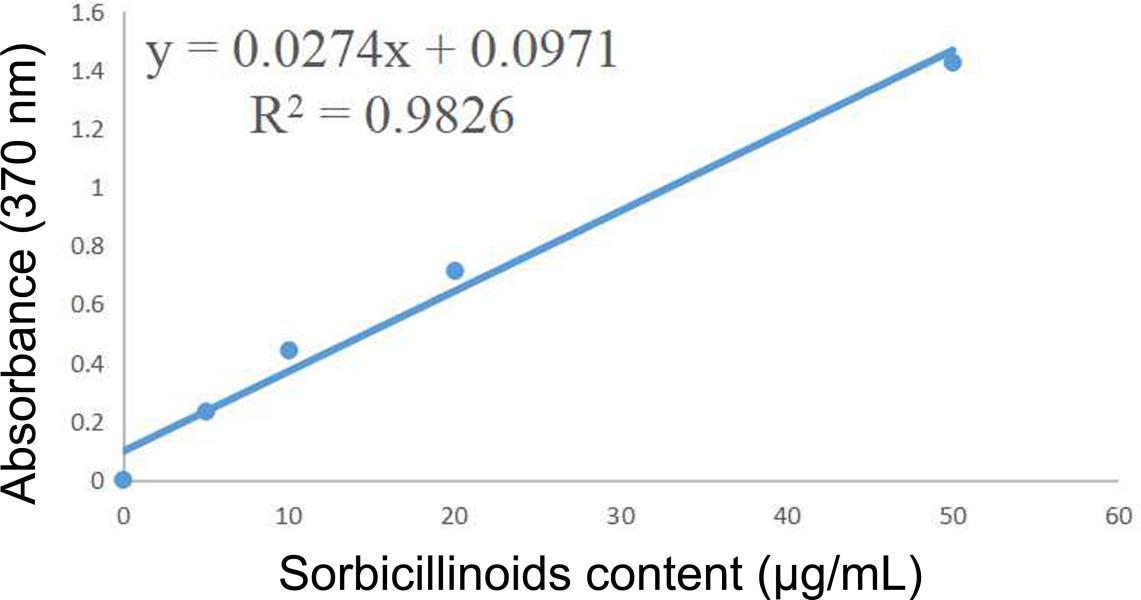


Fig. S3 Thestandard curve of sorbicillinoids.


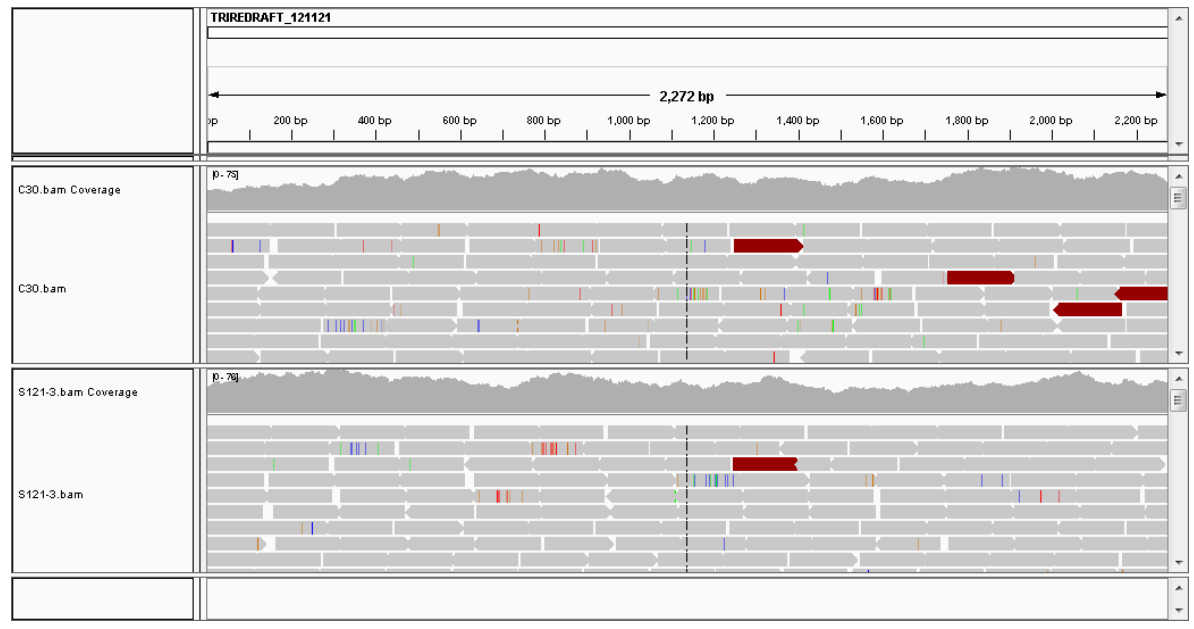


Fig. S4 The whole genome resequencing of strain ZC121 shows the unsuccessful deletion of gene 121121.


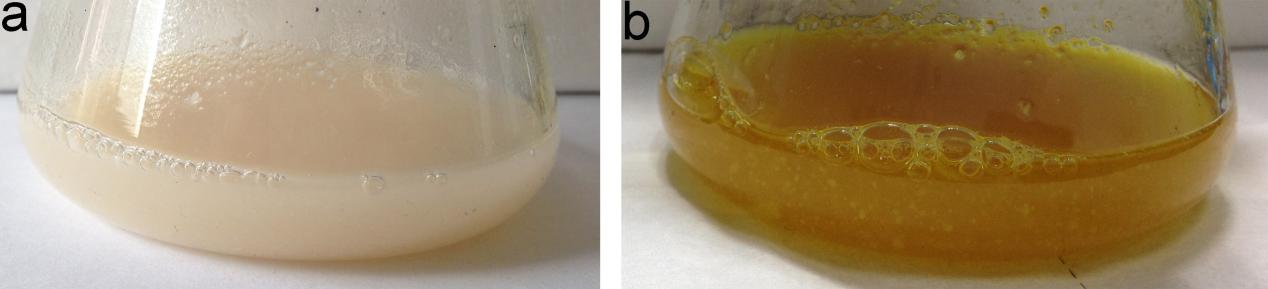


Fig. S5 The color of the culture supernatant of *T. reesei* RUT-C30 (a) and ZC121 (b) grown on glucose.


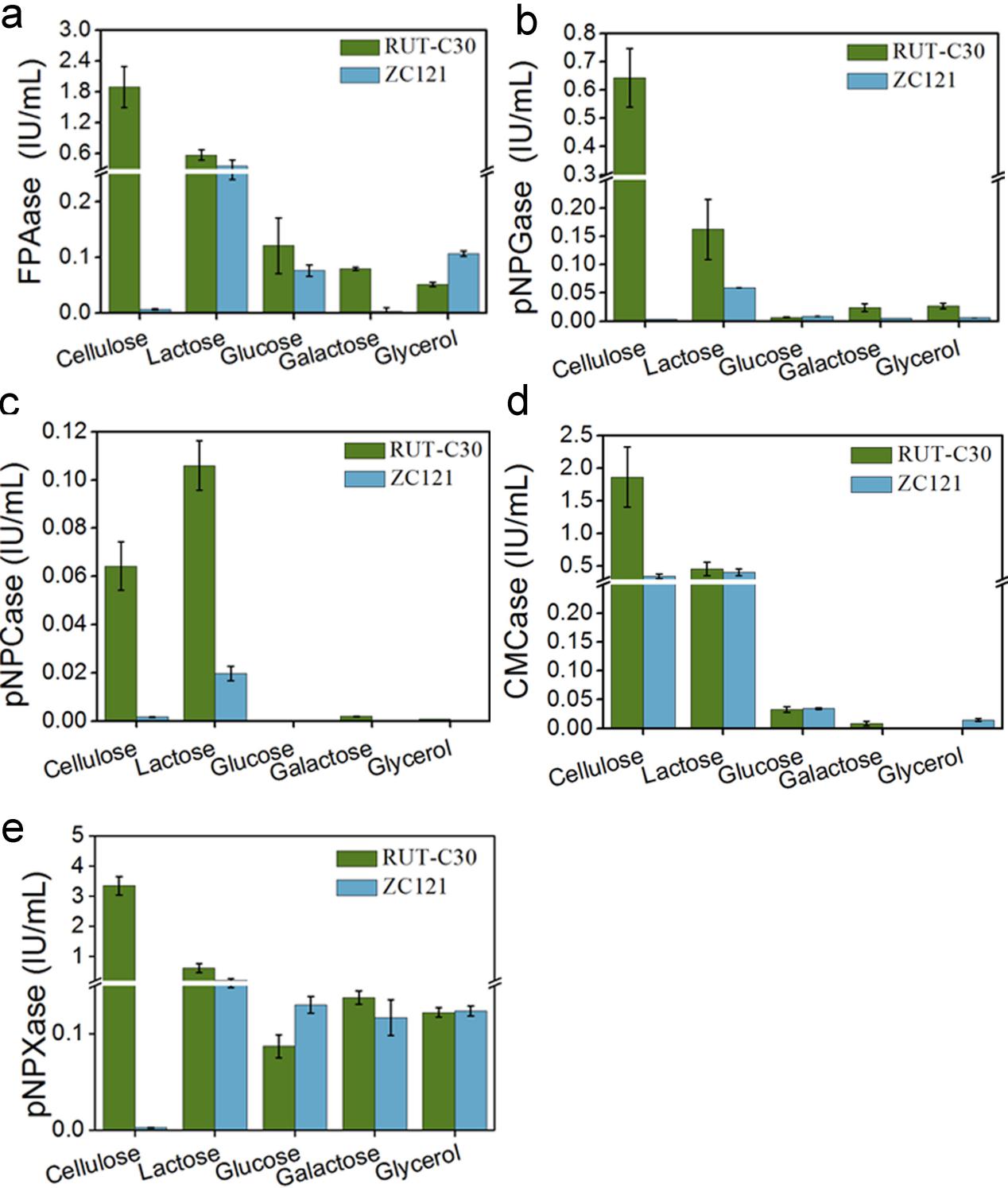


Fig.S6 Cellulolytic enzyme activities in the culture supernatant of *T. reesei* ZC121 and RUT-C30 grown on 2% cellulose, lactose, glucose, galactose and glycerol were assayed on day 5, including the activities of FPase (the filter paper activity) (a), pNPGase (the BGL activity) (b), pNPCase (the CBH activity) (c), CMCase (the CMC activity) (d) ahd pNPXase (the β-xylosidase activity) (e). The error bars indicate the standard deviations of three biological replicates.

Table S1 Primers used in this study.

| Name | Sequence | Description |
| --- | --- | --- |
| 121121(deletion)up-F | ATTATTATGGAGAAACTCGAGCGGAAGCGACGGTGGAGAAGAAAT | Amplification of the 1500 bp upstream of gene 121121 |
| 121121(deletion)up-R | CCGTCACCAGCCCTGCTCGAGTTGTGCTCTAGTGCTGTTGCTGTTCC | Amplification of the 1500 bp upstream of gene 121121 |
| 121121(deletion)down-F | ACGTTATCTCGTGCGGATCCATTAAGTGTATACAGAAGTAGCAGCAC | Amplification of the 1500 bp downstream of gene 121121 |
| 121121(deletion)down-R | CAGGTCGACTCTAGAGAGGATCCGTTCCAGCTCATGGATTACAA | Amplification of the 1500 bp downstream of gene 121121 |
| 121121-(3783bp)-F | ATGGAGACAAACAACAGCGACAGCA | PCR cloning of the 3783 bp fragment as indicated in Figure 1 |
| 121121-(3783bp)-R | GTTCCAGCTCATGGATTACAACAACATCCC | PCR cloning of the 3783 bp fragment as indicated in Figure 1 |
| 121121- (2075bp)-F | CCGCAGGACATATCCACGCCCTC | PCR cloning of the 2075 bp fragment as indicated in Figure 1 |
| 121121- (2075bp)-R | GTTCCAGCTCATGGATTACAACAACATCCC | PCR cloning of the 2075 bp fragment as indicated in Figure 1 |

Table S2 Comparative transcription levels of the sorbicillinoid gene cluster and its neighboring cellulase-related genes in *T. reesei* ZC121on cellulose and glucose.

| Gene ID^a^ | log2(C121/CC30)  Celluose Glucose | | p value  Celluose Glucose | | Gene name | Description |
| --- | --- | --- | --- | --- | --- | --- |
| 67737 | inf | inf | inf | inf | *sor5* | hypothetical protein |
| 93844 | 5.13855 | 7.57345 | 5.00E-05 | 5.00E-05 | *sor1* | hypothetical protein |
| 93847 | 7.24352 | 9.79423 | 5.00E-05 | 5.00E-05 | *sor2* | hypothetical protein |
| 139626 | 11.3587 | 11.6189 | 0.0041 | 5.00E-05 | *sor3* | FAD/NAD(P)-binding domain-containing protein |
| 121436 | 8.76461 | 8.58193 | 5.00E-05 | 5.00E-05 | *sor6* (MFS) | MFS general substrate transporter |
| 31634 | 4.84193 | 5.20684 | 5.00E-05 | 5.00E-05 | *ypr2* | hypothetical protein |
| 4579 | 6.43464 | 5.79129 | 5.00E-05 | 5.00E-05 | *sor4* | putative isoamyl alcohol oxidase |
| 93861 | 4.70987 | 4.77854 | 5.00E-05 | 5.00E-05 | *ypr1* | hypothetical protein |
| 69551 | 4.70987 | 0.00140732 | 5.00E-05 | 0.7632 | 69551 | hypothetical protein |
| 139631 | -5.74706 | -0.736928 | 5.00E-05 | 0.1496 | *axe1* | acetyl xylan esterase |
| 121449 | -6.30054 | -2.7972 | 5.00E-05 | 0.00025 | *cip1* | hypothetical protein |
| 139633 | -5.70598 | -1.33685 | 5.00E-05 | 5.00E-05 | *cel61a* | endoglucanase-4 |

**a:** Gene ID was assigned based on the *T. reesei* RUT-C30genome database (https://www.ncbi.nlm.nih.gov/genome/323?genome_assembly_id=49799). Inf: the transcript of sor5 was not detected in RUT-C30, but in ZC121.
